# Renal Denervation Attenuates Adverse Remodeling and Intramyocardial Inflammation in Acute Myocardial Infarction With Ischemia–Reperfusion Injury

**DOI:** 10.3389/fcvm.2022.832014

**Published:** 2022-04-28

**Authors:** Kun Wang, Yu Qi, Rong Gu, Qing Dai, Anqi Shan, Zhu Li, Chenyi Gong, Lei Chang, Han Hao, Junfeng Duan, Jiamin Xu, Jiaxin Hu, Dan Mu, Ning Zhang, Jianrong Lu, Lian Wang, Han Wu, Lixin Li, Lina Kang, Biao Xu

**Affiliations:** ^1^Department of Cardiology, Nanjing Drum Tower Hospital, The Affiliated Hospital of Nanjing University Medical School, Nanjing, China; ^2^Department of Emergency, Nanjing Drum Tower Hospital, The Affiliated Hospital of Nanjing University Medical School, Nanjing, China; ^3^Department of Cardiology, Nanjing Drum Tower Hospital, Clinical College of Nanjing Medical University, Nanjing, China; ^4^Department of Radiology, Nanjing Drum Tower Hospital, The Affiliated Hospital of Nanjing University Medical School, Nanjing, China; ^5^Department of Ultrasound, Nanjing Drum Tower Hospital, The Affiliated Hospital of Nanjing University Medical School, Nanjing, China; ^6^Physician Assistant Program, The Herbert H. and Grace A. Dow College of Health Professions, Central Michigan University, Mount Pleasant, MI, United States; ^7^State Key Laboratory of Pharmaceutical Biotechnology, Nanjing University, Nanjing, China

**Keywords:** acute myocardial infarction, sympathetic nervous system, renal denervation, cardiac remodeling, inflammation

## Abstract

**Background:**

Inhibition of sympathetic activity and renin–angiotensin system with renal denervation (RDN) was proved to be effective in managing refractory hypertension, and improving left ventricular (LV) performance in chronic heart failure. The inhibition of sustained sympathetic activation prevents or delays the development of cardiac fibrosis and dysfunction that occurs after myocardial infarction and ischemia–reperfusion (I/R) injury. The translational efficiency of RDN remains to be defined in preclinical animal studies.

**Objectives:**

This study investigated the therapeutic role of RDN in adverse remodeling and intramyocardial inflammation in myocardial ischemia–reperfusion (MI/R) injury.

**Methods:**

Herein, 15 minipigs were subjected to 90-min percutaneous occlusion of the left anterior descending artery followed by reperfusion. Eight animals received simultaneous RDN using catheter-based radiofrequency ablation (MI/R-RDN). Cardiac function and infarct volume were measured *in vivo*, followed by histological and biochemical analyses.

**Results:**

The infarct volume in I/R-RDN pigs reduced at 30 days postreperfusion, compared to I/R-Sham animals. The levels of catecholamine and cytokines in the serum, kidney cortex, the border, and infarcted regions of the heart were significantly reduced in I/R-RDN group. Moreover, the gene expression of collagen and the protein expression of adrenergic receptor beta 1 in heart were also decreased in I/R-RDN mice. Additionally, RDN therapy alleviated myocardial oxidative stress.

**Conclusion:**

RDN is an effective therapeutic strategy for counteracting postreperfusion myocardial injury and dysfunction, and the application of RDN holds promising prospects in clinical practice.

## Introduction

Acute myocardial infarction (AMI), the most severe manifestation of coronary artery disease, is one of the leading causes of morbidity and mortality worldwide (1). The early mortality of AMI has decreased dramatically due to timely successful reperfusion therapy with percutaneous coronary intervention (PCI) that protects the heart from permanent damage. However, restoration of blood flow and reoxygenation in the heart is frequently associated with an exacerbation of tissue injury and a profound inflammatory response that is known as ischemia–reperfusion (I/R) injury (2). Myocardial injury induces significant changes in left ventricular (LV) structure which contributes to 75% of AMI survivors who develop heart failure within 5 years (3). The therapeutic approaches focusing on the prognosis of AMI with I/R injury remain to be developed.

It is well established that neurohormonal activation, the overactivation of both sympathetic nervous system (SNS) and renin–angiotensin–aldosterone system (RAAS), as well as an intense sterile inflammatory response are major contributors to reperfusion injury and cardiac remodeling (4). Pharmacotherapies that target the autonomic nervous system improve the prognosis of patients with I/R injury. However, high non-adherence rates limit the optimal use of these drugs due to many adverse effects (5). Novel adjunct cardioprotective maneuvers post-I/R are therefore crucial. Recently, alternative interventional therapeutic treatment, a catheter-based radiofrequency renal denervation (RF-RDN), is already used clinically to reduce SNS and RAAS activation, and seems to be a potential treatment for resistant hypertension (6–9). Furthermore, renal denervation (RDN) treatment exerted a beneficial effect by reducing cardiac remodeling in a porcine model of chronic heart failure (10, 11). Herein, we hypothesize that the function of RDN therapy postreperfusion could limit infarct size and prevent HF development.

Here, a catheter-based bilateral renal sympathetic nerve ablation followed by reperfusion was applied to an established porcine model of AMI with extensive ST-segment elevation myocardial infarction (STEMI). Involvement of both neuronal and humoral pathways allow us to study the signal transfer between renal and heart. We observed that RDN therapy has cardioprotective effects in AMI with I/R injury through improvement in cardiac function and structure. We also found that the protective effect of RDN acts through multiple pathways, including reversing adverse remodeling, decreasing infarct area, reducing neurohumoral changes, and decreasing of inflammation levels.

## Materials and Methods

### Ethics Statement and Animal Preparation

All animal experimental procedures were approved by the Ethics Committee of Nanjing Drum Tower Hospital, The Affiliated Hospital of Nanjing University Medical School (ethics code: 20200508) and conformed to the Guide for the Care and Use of Laboratory Animals according to Chinese National Regulations.

Eighteen 3-month-old Bama miniature pigs, weighing 15 ± 2 kg each, purchased from Taizhou Taihe Biotechnology Co., Ltd., were housed in animal care facilities. Prior to surgery, pigs were fasted for 12 h but with free water. Zoletil^®^ 50 (Virbac) and atropine sulfate were injected intramuscularly for anesthesia, and peripheral venous access was established. Propofol (5 mg/kg/h) was slowly injected intravenously during the operation for general anesthesia. After tracheal intubation to assist breathing, pigs were mechanically ventilated using a non-invasive ventilator (tidal volume limited to 0.4–0.6 L/min, 18 breaths/min, oxygen concentration of 60%). An IntelliVue MP30 electrophysiological recorder was connected to the subjects for continuously dynamically monitoring heart rate, respiration, oxygen saturation, and blood pressure. Randomization was set preoperatively which was blinded to the operator, and when the animals established the acute myocardial I/R, they were grouped with renal denervation **(MI/R-RDN)** or sham **(MI/R-Sham)** procedures accordingly.

### Acute Myocardial Infarction With Ischemia–Reperfusion Injury Minipig Model

**FIGURE 1:**
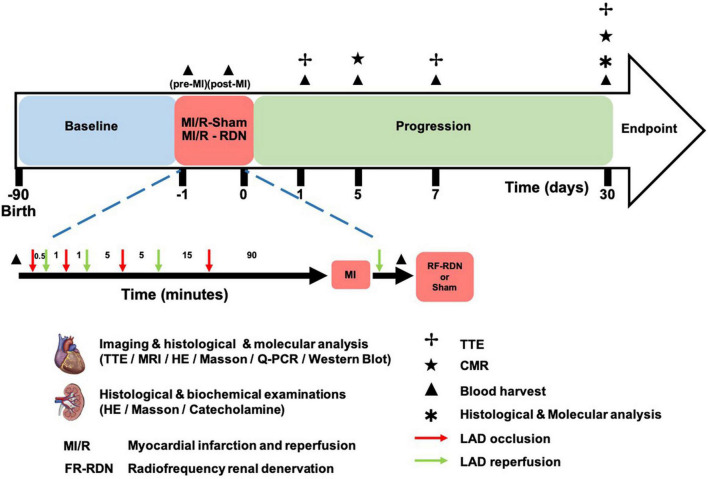
MI/R-RDN experimental protocols. Preconditioned minipigs were subjected to triple episodes of 30 s, 1 and 5 min occlusion followed by 1, 5, and 15 min reperfusion, respectively, before the prolonged ischemia. Then LAD was occluded for 90 min followed by reperfusion before they were immediately treated with sham- or RF-RDN. TTE was performed at 1, 7, and 30 days after RDN therapy. CMR was performed at 5 and 30 days after RDN therapy. Peripheral blood was collected pre-MI/R, post-MI/R, and 1, 5, and 30 days post-MI/R. HE, hematoxylin and eosin; LAD, left coronary artery descending; TH, tyrosine hydroxylase.

**FIGURE 2:**
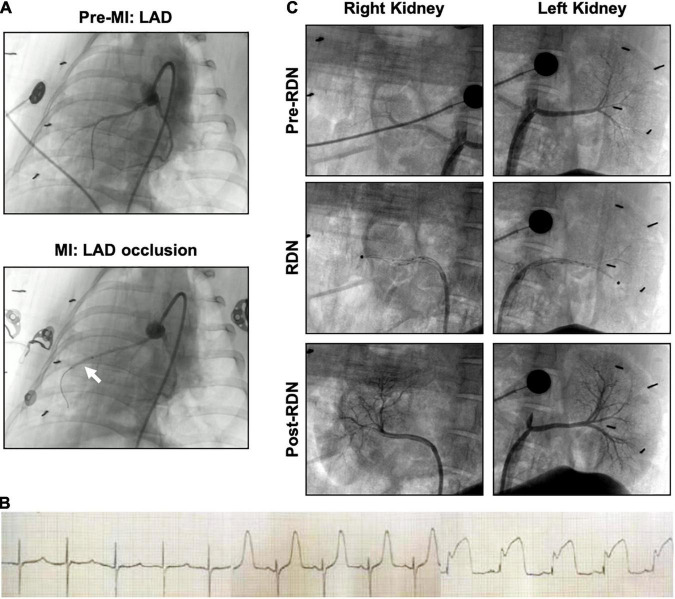
Angiography of acute myocardial infarction and reperfusion (MI/R) and the RDN procedure in the porcine model. **(A)** Coronary artery angiography was performed before and during occlusion. The white arrow indicates the balloon position. **(B)** Representative ECG of the interventional procedure of MI/R. ST segment elevation of the anterior wall lead was observed. **(C)** Renal angiography was performed before, during, and after RDN in a bilateral manner. During the RDN procedure, the ablation catheter (black dot) can be observed in both renal arteries. Lack of stenosis was confirmed post-RDN.

All interventional procedures were performed under general anesthesia and electrocardiographic monitoring. A percutaneous sheath was placed in the femoral artery with a standard sterile technique. An 8F Fast-Cath (St. Jude, United States) was used for LV angiography and heparin (2000 U/h, I.V.) injections, and a 6F EBU3.5 (Medtronic, United States) was placed at the ostia of the left coronary artery descending (LAD). Myocardial ischemia was induced by angioplasty balloon occlusion (2.0 × 15 mm, Maverick, Boston Scientific) with 10 atm. Preconditioned minipigs were successively subjected to triple episodes of 30 s, 1 and 5 min occlusion and 1 min occlusion followed by 5 and 15 min reperfusion, respectively, before the prolonged ischemia. Then LAD was occluded for 90 min followed by reperfusion before they were immediately treated with sham- or RF-RDN (the procedure is shown in Figure 1), and a cine angiogram was performed to confirm total occlusion (shown in Figure 2A). The STEMI model was successfully established when an elevated T wave was observed in the electrocardiogram (ECG) (shown in Figure 2C), and heart rate was recorded (Supplementary Table 1). An external defibrillator was always available after balloon inflation, and it was used appropriately when a pig developed fatal arrhythmias. Eight pigs experienced ventricular fibrillation during blood flow occlusion, seven pigs were rescued successfully, and one pig died without establishment of the model.

### Radiofrequency Renal Artery Denervation

*Via* percutaneous femoral artery access, an 8F renal artery Mach 1 RDC (Boston, Scientific, United States) was placed at the ostia of the renal artery, and then a 12 mm radiofrequency ablation catheter (GL-6W, Shanghai Golden Leaf Medtech Co., Ltd., China) was introduced into the main renal artery. The catheter had six electrodes to sequentially deliver radiofrequency energy, which were mounted on a basket that could be opened to achieve solid contact with the vessel wall. We performed a 120-s ablation with each electrode and two sets of ablations in the renal artery from the ostia to the bifurcation (10–12 total ablations per renal artery), and then repeated the procedure on the other side. The temperature was 60°C. Renal angiography was performed before, during, and after RF-RDN to confirm patency of the arteries (shown in Figure 2B). The MI/R-Sham group procedure involved the same guide catheter and ablation catheter placement without activation of the RF generator.

Benzylpenicillin was administered intramuscularly for two consecutive days to prevent postoperative infection. Two pigs died after the operation: one in the MI/R-Sham group died within 24 h after the surgery, and the other in the MI/R-RDN group died during the first magnetic resonance imaging scanning due to the anesthetic overdose.

### Transthoracic Echocardiography

Two-dimensional transthoracic echocardiography (TTE) was performed with a CX-50 ultrasound system (Phillips, Amsterdam, Netherlands) equipped with an S5-1 array sector transducer probe at a frequency range of 3.5–5.5 MHz. To assess cardiac function, echocardiographic examinations of each minipig were performed under anesthesia to produce quality images at 1, 7, and 30 days post-myocardial ischemia–reperfusion (MI/R). Left ventricular diameter at end-systole or end-diastole (LVESD, LVEDD), interventricular septal thickness (IVS), and left ventricular posterior wall thickness (LVPW) were measured from M-mode in parasternal short-axis or long-axis view at a level close to the papillary muscles. Three to six representative contraction cycles were used for analyses, and the left ventricular ejection fraction (LVEF) was calculated using the biplane Simpson method with the accompanying software. LV mass was calculated according to the recommendations of the American Society of Echocardiography and the European Association of Cardiovascular Imaging. LV diastolic function was evaluated using the ratio of early transmitral flow velocity (E) to late transmitral flow velocity (A) and the mean of transmitral E to early diastolic medial LV tissue velocity of the lateral wall (e′ lateral). The velocity-time integral (VTI) of the left ventricular outflow tract (LVOT) was obtained from pulsed Doppler imaging by positioning the sample volume at the LVOT approximately 0.5 cm below the aortic valve. Two blinded examiners performed all measurements.

### Cardiac Magnetic Resonance Imaging

Cardiac magnetic resonance imaging (CMR) was performed to assess the infarct area and cardiac function 5 and 30 days after the MI/R-Sham or MI/R-RDN operation using an Ingenia CX 3.0T system (Philips Healthcare, Best, Netherlands). Steady-state free-precession cine imaging was performed using a 32-element phased-array body coil with ECG gating in cardiac vertical and horizontal short-axis and long-axis orientations.

The following scanning parameters were used: time of repetition (TR), 3.5 ms; time of echo (TE), 1.72 ms; field of view (FOV), 320 mm × 320 mm; layer thickness, 8 mm; the number of layers in the LV short axis, 8; the number of layers in the rest of the image, 3; flip angle (FA), 45°; and the number of excitations, 30 cardiac cycles. Ten to 15 min after the contrast agent injection (gadodiamide, 0.1 mmol/kg), a T1-weighted segmented phase-sensitive inversion recovery gradient-echo sequence was acquired for cine images to detect late gadolinium enhancement (LGE). The following scanning parameters were used: TR, 5.1 ms; TE, 2.5 ms; FOV, 255 mm × 255 mm; layer thickness, 8 mm; the number of layers in the LV short axis, 8; the number of layers in the rest of the image, 3; FA, 45°; and the number of excitations, 30 cardiac cycles. The infarct area was assessed as the area of hyperenhancement on the LGE images, which was measured as absolute mass or as a percentage of the entire LV myocardial mass. The images were analyzed by two independent investigators with software (QMass MR 7.5, Medis, Netherlands) according to the manufacturer’s instructions.

### Catecholamine Measurements

Peripheral blood samples were collected at baseline, post-MI, and 1, 7, and 30 days post-RDN. Renal cortex tissue and myocardial tissue were harvested at 30 days post-RDN, immediately frozen in liquid nitrogen, and stored at −80°C until catecholamine assays were performed. Portions of tissue from the necrotic, border zone (BZ), and remote zone (RZ) of the heart and right and left kidney cortex were mechanically homogenized in 10 volumes of ice-cold NaCl using a homogenizer. Plasma and tissue homogenates were assayed using high-performance liquid chromatography (HPLC, Thermo Scientific UltiMate 3000). Analytical run times of norepinephrine, adrenaline, and dopamine were 3.5, 3.95, and 8.10 min, respectively. The data are graphed as nanograms of analyte per gram (ng/g) of total tissue.

### ELISA and Cardiac Troponin T Detection

Blood samples were collected and centrifuged at 3000 rpm for 20 min. Serum was collected to measure the plasma levels of tumor necrosis factor-alpha (TNF-α) (R&D, PTA00) using ELISA kits according to the manufacturer’s protocol. High-sensitivity cardiac troponin T (cTnT) (Roche Diagnostics) was measured on Cobas e411 and i1000SR analyzers using the manufacturer’s calibrators and quality controls (12). The detection limit of the cTnT assay was set to 3 ng/L by the manufacturer.

### Oxidative Stress Measurements

Malondialdehyde (MDA) and superoxide dismutase (SOD) levels in LV BZ tissues were measured using kits (Jiancheng Bio, China) according to the manufacturer’s instructions.

### Histological Staining

Thirty days after reperfusion, pigs were euthanized with potassium chloride (40 mEq/kg, I.V.). Tissues, including renal arteries and hearts, were collected for histological, biochemical, and molecular analyses. The renal arteries and surrounding tissues were subjected to immunohistochemical staining for hematoxylin and eosin (HE) and tyrosine hydroxylase (TH, Abcam, ab41528, 1:1000). Briefly, renal artery segments were harvested and fixed in 4% paraformaldehyde for 24 h, washed and placed in 70% ethyl alcohol until tissue processing for paraffin embedding. Renal artery samples were embedded and sliced into 2 μm thick serial cross-cryosections. LV tissue isolated from the infarct zones (IZs), BZs, and RZs was cut into 1 cm sections and 5 μm cross-sections were mounted onto glass slides and stained with HE and Masson’s trichrome staining for analysis of the infarct area and fibrosis. CD163 (Proteintech, 16646-1-AP, 1:200) were stain for inflammatory cells under established protocol.

### RNA Extraction and Quantitative Real-Time Polymerase Chain Reaction

The LV IZ, BZ, and RZ were lysed with RNAiso plus Reagent (Takara, 9109) according to the manufacturer’s instructions, and total RNA was extracted. Purified RNA was quantified, and cDNA was synthesized using SuperMix (Vazyme, R223) and amplified with SYBR Green Master Mix (Vazyme, Q711) in a Quant Studio 6 Flex Real-Time PCR System (Applied Biosystems). GAPDH was used as a housekeeping gene, and the 2^–ΔΔ*CT*^ formula was used for data analysis. The primers sequences are shown in Supplementary Table 2.

### Western Blot

Heart tissue was quickly removed and immediately homogenized in EDTA-free RIPA buffer (Cat No. P0013B, Beyotime) at 4°C. After protein concentration measurement, the lysate was stored at −80°C for Western blot analysis. The lysate protein (20 μg) was electrophoresed and transferred to polyvinylidene difluoride (PVDF) membranes. After blocking with 5% non-fat dry milk, the membrane was incubated with primary antibodies at 4°C overnight. The following antibodies were used: beta 1 adrenergic receptor (Invitrogen, PA5-28808, 1:2000), and GAPDH (Proteintech, 10494-1-AP, 1:5000). The next day, membranes were incubated with secondary antibodies at RT for 1 h, followed by protein detection with SuperSignal West Femto Maximum Sensitivity Substrate (Thermo Fisher, 34096). Total protein expression was normalized to GAPDH.

### Statistical Analysis

Data were statistically analyzed using GraphPad Prism 8.4.0 software with Student’s unpaired *t*-test for two-tailed comparisons at a single time point and two-way analysis of variance with Bonferroni correction to account for multiple comparisons. All data are presented as the mean ± standard error of the mean (SEM), and a (corrected) *p*-value less than 0.05 was indicative of statistically significance.

## Results

### Validation of Acute Myocardial Infarction and Reperfusion Porcine Model

Representative angiographic heart images before and during the induction of MI with balloon occlusion are showed in Figure 2A. The white arrow indicates the balloon position. The STEMI in the ECG indicated that the AMI model was successfully established (Figure 2B). The time and the extent of ischemic injury were similar in both groups, as demonstrated by ECG. Eight minipigs experienced ventricular fibrillation after balloon occlusion, seven minipigs were rescued successfully, and one pig died. Besides, two pigs died after the operation: one died within 24 h of AMI model establishment, and the other died during CMR scanning due to anesthetic overdose.

### Validation of Catheter-Based Renal Sympathetic Denervation Effectiveness

**FIGURE 3:**
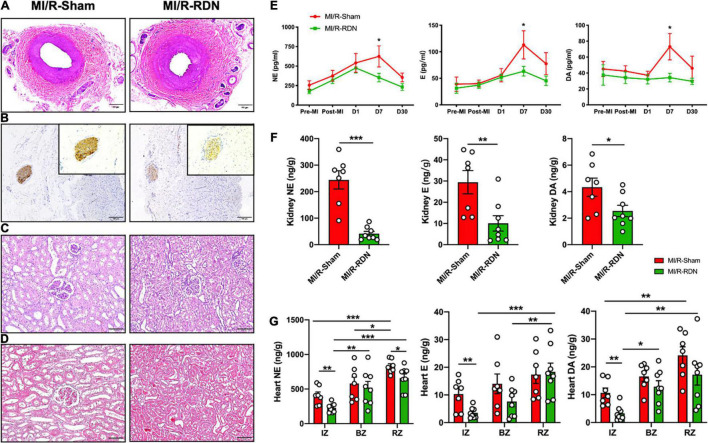
Histological staining of the kidneys and renal arteries and catecholamine levels in the circulation, renal cortex, and heart. **(A)** Representative images of hematoxylin and eosin (HE) staining of the renal arteries at the 30-day endpoint following sham or RDN therapy. **(B)** Representative images of tyrosine hydroxylase (TH) staining on the nerves in the adventitial layer of the renal arteries at the 30-day endpoint following sham or RDN therapy. Inserts indicate renal artery nerves and magnified photomicrographs (20×) of TH staining. The scale bar is 100 mm. **(C)** Representative images of HE staining of the kidney at the 30-day endpoint following sham or RDN therapy. The scale bar is 100 mm. **(D)** Representative images of Masson’s trichrome staining of the kidney at the 30-day endpoint following sham or RDN therapy. The scale bar is 100 mm. **(E)** Norepinephrine (NE), epinephrine (E), and dopamine (DA) concentrations in the circulation pre-AMI, post-AMI, and 1, 7, and 30 days post-RDN. **(F)** NE, E, and DA concentrations per gram of kidney cortex tissue. **(G)** NE, E, and DA concentrations per gram of heart IZ, BZ, and RZ tissue. *n* = 7 in the IM/R-Sham group, *n* = 8 in the IM/R-RDN group. IZs, infarct zones; BZs, border zones; and RZs, remote zones. Data are shown as the mean ± SEM **(E–G)**. *p*-Values were obtained by two-way ANOVA with Bonferroni test **(E)**, unpaired, two-tailed *t*-test **(F)**, and two-tailed *t*-test and one-way ANOVA with Bonferroni test **(G)**. *p < 0.05, **p < 0.01, and ***p < 0.001 (compared with the MI/R-Sham or MI/R-RDN group).

Representative pre-, during-, and post-RDN angiographic renal images confirmed patency of the arteries (Figure 2C). The locations of ablation delivery are indicated by small black points in the vessels. Thirty days after RDN therapy, renal arteries were collected and sectioned for HE and TH staining to assess nerve viability and the catecholamine production. TH is the rate-limiting enzyme in catecholamine biosynthesis, and is expressed primarily in the perivascular area. Representative photomicrographs illustrated the reduced TH staining in the MI/R-RDN group compared to the MI/R-Sham group (Figures 3A,B). Furthermore, to assess kidney structure, HE and Masson’s trichrome staining were also performed and showed no changes (Figures 3C,D). These data clearly demonstrated that the RDN procedure reduced catecholamine production from the renal sympathetic nerves without impairing renal artery or kidney structure.

### Renal Denervation Therapy Affects Catecholamine Levels

Peripheral blood samples were obtained at baseline, post-MI, and 1, 7, and 30 days post-RDN. The renal cortex was obtained at 30 days post-RDN. Serum samples and kidney cortex tissues from the MI/R-RDN and MI/R-Sham groups were analyzed using liquid chromatography–mass spectrometry (LC/MS). Serum dopamine (DA), adrenaline (E), and norepinephrine (NE) levels were significantly reduced at 7 days post-RDN but not at 30 days post-RDN (Figure 3E). Kidney NE, E, and DA levels were significantly reduced in the MI/R-RDN group (Figure 3F). The catecholamine concentrations in the heart were evaluated and revealed that NE, E, and DA levels from the IZs were significantly reduced. While heart NE levels also decreased in the RZ, other indicators remained unchanged in the BZs and the RZ (Figure 3G).

### Cardiac Magnetic Resonance Imaging Indicated That Renal Denervation Inhibits Left Ventricular Remodeling

**FIGURE 4:**
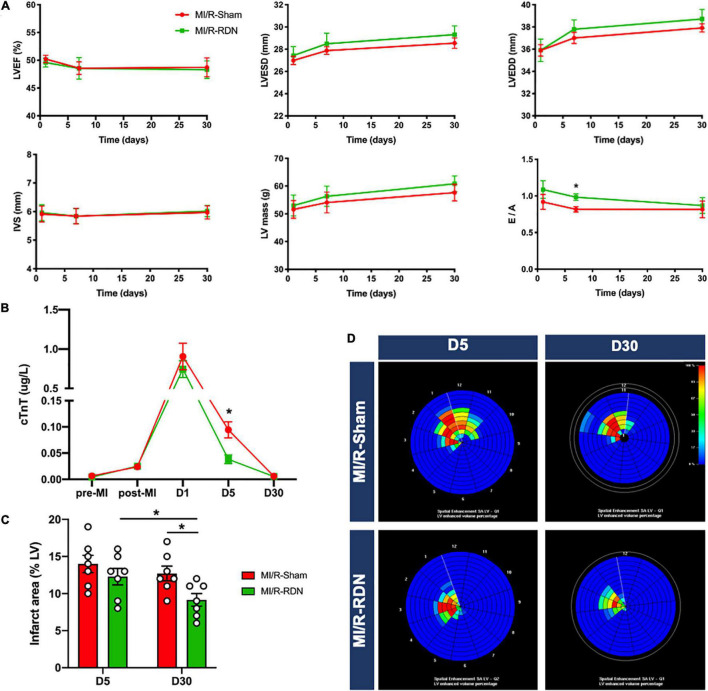
Echocardiography and CMR outcomes. **(A)** The parameters of echocardiography, left ventricular ejection fraction (LVEF), interventricular septal thickness (IVS), LV mass, and the ratio of early transmittal flow velocity to late transmittal flow velocity (E/A) were measured with TTE (*n* = 7 each group). **(B)** Serum cardiac troponin T (cTnT) was measured pre-AMI, post-AMI, and 1, 5, and 30 days post-RDN (*n* = 7 each group). **(C)** Analysis of the percentage of the enhanced volume (*n* = 7 each group). **(D)** Representative graphs of the percentage of the enhanced volume assessed using CMR. Data are shown as the mean ± SEM **(A–C)**. *p*-Values were shown and assessed by two-way ANOVA with Bonferroni test **(A–C)**. *p < 0.05 (compared with the MI/R-Sham group).

To assess cardiac contractile function, we measured LV dimensions and volumes using TTE. There were no changes in LVEF, LVESD, LVEDD, LV mass, or IVS between the groups at 1, 7, and 30 days post-RND. However, diastolic function was improved at 7 days in the MI/R-RDN group compared to the MI/R-Sham group (Figure 4A). In addition, serum cTnT levels, which evaluate myocardial injury after MI/R injury, were increased in all pigs. Interestingly, the cTnT level did not reach a significant threshold 24 h post-RDN between the two groups, but showed a lower level at 5 days in the MI/R-RDN group (Figure 4B). Furthermore, to verify whether RDN effectively treated cardiac remodeling in MI/R, we performed experiments with CMR using LGE at 5 and 30 days post-RND. The infarct area (percentage of the enhanced volume) was similar between the two groups at 5 days. However, it was significantly decreased in the MI/R-RDN group at 30 days compared to the MI/R-Sham group (Figures 4C,D).

### Renal Denervation Therapy Attenuates Oxidative Stress and Fibrosis

**FIGURE 5:**
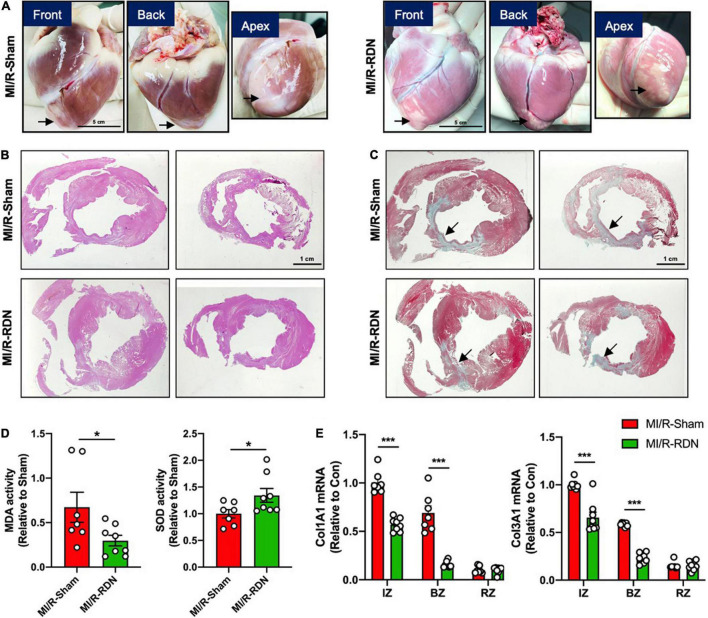
Histological analysis and collagens expression levels. **(A)** Minipig heart tissue was taken from the front, back, and apex. **(B)** Representative photomicrographs of HE-stained heart sections from the MI/R-sham- and MI/R-RDN-treated animals. **(C)** Representative photomicrographs of Masson’s trichrome-stained heart sections from the MI/R-sham- and MI/R-RDN-treated animals. **(D)** Activity of the malondialdehyde (MDA) and the antioxidant enzyme superoxide dismutase (SOD) in the border zone of LV were detected. The data are shown as the fold change relative to the MI/R-Sham group. **(E)** Fibrotic gene profile consisting of the mRNA expression of collagen 1 (Col1A1) and collagen 3 (Col3A1). The expression of these genes was normalized to GAPDH expression and is shown as the fold change relative to the IZ in the MI/R-Sham group. *n* = 7 in the IM/R-Sham group, *n* = 8 in the IM/R-RDN group. Data are shown as the mean ± SEM **(D,E)**. *p*-Values were shown and assessed by unpaired, two-tailed *t*-test **(D)** and two-way ANOVA with Bonferroni test **(E)**. *p < 0.05, ***p < 0.001 (compared with the MI/R-Sham group).

Ventricular morphology was assessed with HE staining, and collagen content was quantified using Masson’s trichrome staining (Figures 5A–C). LV homogenates from the BZ were analyzed to quantify the levels of oxidative stress at 30 days post-RDN. As depicted in Figure 5D, myocardial oxidative stress (MDA levels) was reduced in the MI/R-RDN group compared to the MI/R-Sham group. Furthermore, the protein expression of antioxidant enzyme (SOD) was upregulated after RDN therapy (Figure 5D). Gene expressions of collagen 1 and 3, as fibrotic proteins comprising approximately 90% of all cardiac collagens, were significantly reduced in the IZ and BZ but not in the RZ (Figure 5E). Taken together, these results indicated that immediate RDN therapy after reperfusion attenuated cardiac oxidative stress levels and significantly decreased fibrosis at the infarcted and border sites.

### Sympathetic Activity and Myocardial Inflammation Outcome

**FIGURE 6:**
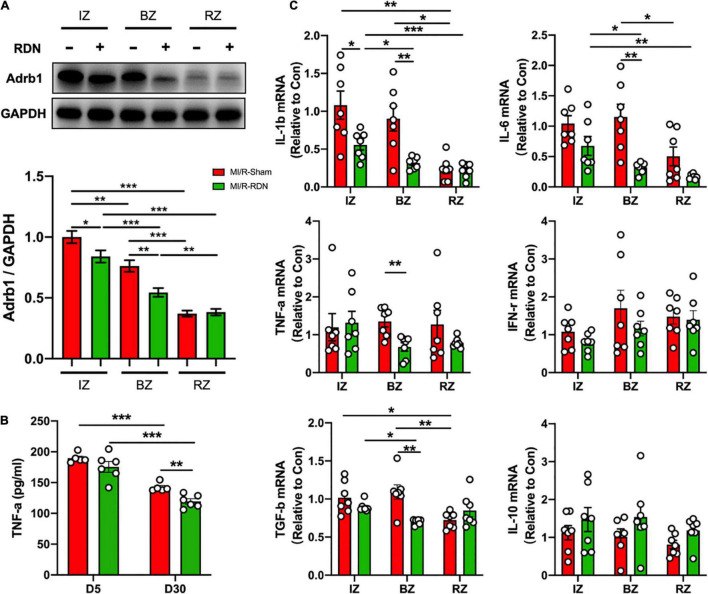
Sympathetic activity and inflammation level in the LV IZ/BZ/RZ in MI/R following with sham or RDN treatment. **(A)** Western blots and statistics of the expression of adrenergic receptor beta 1 (Adrb1) in the infarct zones (IZs), border zones (BZs), and remote zones (RZs) of heart tissues (*n* = 7 each group). **(B)** TNF-α levels in the circulation at 5 and 30 days post-RDN therapy were measured using ELISA (*n* = 5 in the IM/R-Sham group, *n* = 6 in the IM/R-RDN group). **(C)** mRNA levels of inflammatory mediators in heart tissues. The expression of these genes was normalized to GAPDH expression and is shown as the fold change relative to the IZ in the MI/R-Sham group (*n* = 6–7 each group). Data are shown as the means ± SEM. *p*-Values were shown and assessed by two-way ANOVA with Bonferroni test **(A–C)**. *p < 0.05, **p < 0.01, and ***p < 0.001 (compared with the MI/R-Sham group).

Sustained sympathetic signaling results in overactivation of beta-adrenergic receptor (Adrbs) and triggers deranged neural-inflammation circuits that lead to exacerbation of myocardial injury (13). We next examined whether RDN attenuates adrenergic activation in the heart. Notably, the protein expression of adrenergic receptor beta 1 (Adrb1) was significantly decreased in the IZ and BZ but not in the RZ with RDN therapy compared to sham treatment (Figure 6A). The highly prevalent nature of systemic and cardiac inflammation, as a common pathobiological feature, was linked to the development, progression, and complication of poor outcomes of AMI-reperfusion injury. To address the possibility that RDN attenuates inflammation, we performed ELISA assay on serum samples collected on days 5 and 30 after MI/-RDN and quantitative real-time polymerase chain reaction (Q-PCR) on heart tissue samples derived from the IZ, BZ, and RZ collected on day 30 after MI/-RDN. The circulating TNF-α level was significantly decreased in the MI/R-RDN group at day 30 (Figure 6B). Cytokines, including proinflammatory signaling IL-1β, IL-6, TNF-α, and myocardial fibrosis signaling TGF-β, were significantly reduced in the BZ. However, only IL-1β was decreased in the IZ and no significant difference in INF-γ level or proinflammatory resolving lipid mediator IL-10 mRNA levels were detected in the IZ and BZ (Figure 6C). These results suggested that RDN treatment had a beneficial effect of on heart inflammation.

## Discussion

The main findings of our preclinical work are that RF-RDN therapy reduces catecholamine levels, improves adverse cardiac remodeling, decreases infarct volumes and inhibits inflammation in an AMI/reperfusion minipig model. Taken together, our results indicate that RF-RDN is a simple, fast, and effective intervention therapy that allows us to address the crosstalk between the renal sympathetic system and the heart adrenergic system. Therefore, RDN is a potential supplementary therapy for AMI after revascularization.

In the current study, the STEMI model was established by performing increasing episodes before reaching the index 90 min occlusion. Ischemic preconditioning could protect heart from a subsequent prolonged period of ischemia and offer one of the most powerful mechanisms for reducing the speed and extent of myocardial cell damage and the occurrence of malignant arrhythmia in an acute or sustained ischemic insult (14, 15). Hence, it is highly possible that the rapid adaptation to ischemia protects the ischemic heart against a following prolonged ischemic periods and eventually improves pig survival.

The beneficial role of RDN in attenuating ventricular remodeling was confirmed in current study through assessing cardiac function and structure using cardiac TTE and CMR techniques. Clinical guidelines recommend TTE as the first-line diagnostic modality for the evaluation of cardiac diseases. Interesting results showed that at post-RDN day 7, there was an improvement in the E/A. Nevertheless, neither LVEF nor LV mass showed differences at post-RDN day 30 between the I/R-RDN group and the I/R-Sham group. This may due to the low value of LVEF at baseline resulting in no significant difference after the MI operation. CMR is well accepted as the gold standard for the quantification of infarct area (16–18). Our results showed that at 30 days post-RDN, the percentage of infarct size was significantly decreased in the MI/R-RDN group compared with the MI/R-Sham group, indicating that RDN therapy promotes faster repair of the myocardium.

Acute myocardial infarction results in a severe imbalance of metabolic supply and demand of oxygen and nutrients, and leading to tissue hypoxia, which initiates a primary pathophysiological process that is compensated by SNS and RAAS activation (19). Longstanding high levels of NE released from SNS induce changes in cardiomyocyte phenotype by reducing the oxygen supply and increasing the oxygen demand, which contribute to the progression of LV remodeling and myocardial stunning. Recently, Polhemus et al. observed that RDN exhibited cardioprotective actions in the chronic hypertensive SHRs (20). Sharp et al. investigated that RDN prevented heart failure progression via inhibition of renal NE and circulating angiotensin I and II expression in Yucatan minipigs (11), indicating that RDN reduces catecholamine levels in the kidney instead of heart tissue. However, the underlying mechanism of how RDN protects against heart failure progression has not been identified (16). Furthermore, the signal transduction of RDN in cardiac remodeling after AMI, especially reperfusion, was not reported in detail in previous studies. In this study, we founded that serum DA, E, and NE levels were significantly reduced at 7 days with RND therapy, while catecholamine levels downregulation in both kidney cortex and heart tissues at 30 days post-RDN (Figure 3). These results showed that RDN therapy had the potential to attenuate cardiac catecholamine levels.

Catecholamines enhance the mechanical performance of the heart by activating cardiac Adrb. Although cardiomyocytes coexpress β1 and β2, β1 is the predominant subtype and principal driver of catecholamine-driven sympathetic responses in the healthy heart. We examined the protein expression of Adrb1 and observed a significantly reduction in the IZ and BZ of the hearts with RDN therapy but not in the RZ. These results indicated that RDN attenuated only overactivated SNS, but had no obvious inhibitory effect on the normal SNS. Besides, immune cells also express Adrbs. So, we performed the histological staining of CD168 for inflammatory cells staining (Supplementary Figure 1), which showed less numbers of CD168 positive cells in the BZ in the MI/R-RDN group compared with the MI/R-Sham group (data not show). Thus, the lower Adrb1 expression detected in heart tissue cannot be ruled out as a result of reduced inflammatory cell recruitment and infiltration. Considering the heterogeneity and plasticity of immune cells, we will further explore immune cell dynamic balance and molecular mechanisms in myocardial injury and repair in the future. In addition, we observed that RDN reduced heart rate slightly, although there was no difference between I/R-RDN group and I/R-Sham group (Supplementary Table 1). Notably, RDN did not introduce hypotension, which indicated that RF-RDN was safe for the acute hemodynamic changes during the myocardial infarction/reperfusion period.

The proinflammatory cytokines TNF, TL-1β, and IL-6 are markedly and consistently increased in experimental models of AMI or I/R injury and have provided important insights into the mechanisms of inflammation-induced adverse LV remodeling, which may be mediated by uncoupling of Adrb and impaired calcium cycling (17, 18, 21). Our data suggested that RDN treatment leads to a significant reduction on cardiac inflammatory cytokines, including TNF-α, IL-1β, IL-6, and TGF-β. Furthermore, circulating TNF-α levels were also decreased in the MI/R-RDN group. The TNF-α concentration is an independent predictor of mortality and has emerged as a potential therapeutic strategy in AMI and HF. As a highly pleiotropic mediator, TNF-α also protects cardiomyocytes from apoptosis. Excessive TNF-α expression and subsequent activation of cardiomyocyte TNF receptor type 1 induces cardiac contractile dysfunction, hypertrophy, and fibrosis, while a lower TNF-α concentration and subsequent activation of cardiomyocyte TNF receptor type 2 induces a cardiac protective effect (21). However, based on the results from two large clinical trials, high-intensity TNF-α blockade therapy did not improve outcomes in chronic HF patients (22, 23), suggesting that TNF-α may function as a biomarker for anti-inflammatory effects rather than an appropriate therapeutic target. Treatment with an anti-IL-6 receptor antibody not only attenuated adverse remodeling in an infarct mouse model (24) but also increased myocardial salvage in patients with acute STEMI (25). Therefore, timely downregulation of IL-6 levels might be crucial for infarct healing. The TGF-β signaling cascade is a key molecular link between the inflammatory and reparative pathways and regulates the fibrogenic response in the remodeling myocardium (26). Neurohumoral mediators, such as aldosterone, are important for fibroblast activation (27), thus their effects might be mediated in part through activation of TGF-β signaling. Therefore, anti-inflammatory and anti-fibrosis are important therapeutic strategies for improving outcomes following I/R injury.

Infarction and necrosis trigger a strong inflammatory response that induces endothelial cell adhesion molecule synthesis and leads to the recruitment of inflammatory cells to clear the wound of dead cells or matrix debris (28). The spleen as a reservoir for inflammatory cells, plays a central role in the systemic immune response (26). Adrenoceptors are involved in the mobilization of leukocytes from the spleen (29). Splenic activation regulated by SNS, boosts extramedullary hematopoiesis and contributes to a sustained increase in monocyte/macrophage infiltration following I/R injury. The role of the spleen in I/R injury was also demonstrated by positron emission tomography imaging of splenic fluor-deoxyglucose uptake in patients with acute syndrome and at postmortem autopsy (30, 31). Recent evidence suggests the cardioprotection effect of the spleen in remote ischemic preconditioning in pigs and rats model after the denervation experiments, but fail to distinguish between parasympathetic and sympathetic innervation in that study (32). Thus, there is a crosstalk between the splenic immune cells mobilization and cardiac inflammation, and whether RDN can regulate this interplay remains to be further explored.

## Conclusion

Taken together, our data demonstrate that RDN ameliorates oxidative stress, neurohormonal activation, adverse LV remodeling, and inflammation in a large animal model of AMI with I/R injury. These results provide strong evidence of the feasibility and therapeutic efficacy of RDN and suggest the necessity for further development of this therapeutic modality.

## Study Limitations

There are some limitations of our study. First, hemodynamic evaluations of parameters, such as the mean aortic pressure, were not recorded but no intraoperative hypotension was observed during the study. Second, circulating IL-1 and IL-6 levels after MI were below detection range in our model. Therefore, we were unable to assess the direct effect of RDN on systemic cytokines level. In addition, the long-term effects of RDN and its ability to treat complex models in clinical practice should be evaluated. Although, the severity and mortality of MI are higher in female patients regardless of age, our study did not evaluate gender differences and only female animals were used in the present study. Besides, this mice strain is sexually mature at 3 months of age. In order to compare with other miniature pig strains, the weight somewhat limits the age range. Last but not least, the lower Adrb1 expression detected in heart tissue cannot be ruled out as a result of reduced inflammatory cell recruitment and infiltration. Considering the heterogeneity and plasticity of immune cells, we will further explore their dynamic balance and molecular mechanism in myocardial injury and repair in the future.

## Perspectives

### Clinical Competencies

Renal denervation treatment attenuated cardiac remodeling and intramyocardial inflammation by inhibiting sympathetic activity in a porcine AMI with I/R injury model, which is similar to pharmacotherapies that target the autonomic nervous system to improve the prognosis.

### Translational Outlook

Understanding the cardioprotective effects of RDN therapy in AMI with I/R injury is essential in developing clinical management strategies that is in conjunction with primary PCI for STEMI patients to prevent late-onset heart failure.

## Data Availability Statement

The raw data supporting the conclusions of this article will be made available by the authors, without undue reservation.

## Ethics Statement

The animal study was reviewed and approved by the Ethics Committee of Nanjing Drum Tower Hospital, The Affiliated Hospital of Nanjing University Medical School (20200508).

## Author Contributions

LK and BX conceived and designed the work. KW, LK, YQ, and QD performed the PCI and RDN surgeries. All co-authors participated in the material preparation, molecular experiments, and data collection. YQ, KW, and RG performed the data analyses. DM assisted the interpretation of CMR measurement data. NZ blindly performed the echocardiogram examination and statistically analyzed the data. YQ and KW wrote the first draft of the manuscript. BX, LK, LL, and RG revised the manuscript critically for important intellectual content. All authors read and approved the final manuscript.

## Conflict of Interest

The authors declare that the research was conducted in the absence of any commercial or financial relationships that could be construed as a potential conflict of interest.

## Publisher’s Note

All claims expressed in this article are solely those of the authors and do not necessarily represent those of their affiliated organizations, or those of the publisher, the editors and the reviewers. Any product that may be evaluated in this article, or claim that may be made by its manufacturer, is not guaranteed or endorsed by the publisher.

## Abbreviations

Adrb1: adrenergic receptor beta 1
BZs: border zones
ECG: electrocardiogram
IVS: interventricular septal thickness
IZs: infarct zones
LAD: left atrial diameter
LC/MS: liquid chromatography–mass spectrometry
LGE: late gadolinium enhancement
LVEF: left ventricular ejection fraction
MDA: malondialdehyde
MI/R: myocardial ischemia–reperfusion
CMR: cardiac magnetic resonance imaging
NE: norepinephrine
PCI: percutaneous coronary intervention
RAAS: renin–angiotensin–aldosterone system
RF-RDN: radiofrequency renal denervation
RZs: remote zones
SNS: sympathetic nervous system
SOD: superoxide dismutase
STEMI: ST-segment elevation myocardial infarction
TH: tyrosine hydroxylase
TNF-α: tumor necrosis factor-alpha
TTE: transthoracic echocardiography.
