# Supplementary material for: Renal Denervation Attenuates Adverse Remodeling and Intramyocardial Inflammation in Acute Myocardial Infarction With Ischemia–Reperfusion Injury
Source: Front Cardiovasc Med. 2022 Apr 28;9:832014. doi: 10.3389/fcvm.2022.832014 (PMC9095912; doi:10.3389/fcvm.2022.832014)
Supplement: Supplementary file 2 [file Data_Sheet_2.pdf]

Supplemental Materials

Supplemental Table 1. Heart rate measurements during the operation.

HR (bpm)	MI/R-Sham (n = 7)	MI/R-RDN (n = 8)	P value
Baseline	116 ± 15	116 ± 16	0.9769
Occlusion 30s	129 ± 18	130 ± 15	0.9359
Occlusion 1min	129 ± 16	124 ± 12	0.5410
Occlusion 5min	120 ± 13	115 ± 22	0.6356
Occlusion 90min	101 ± 16	100 ± 13	0.8778
Post-Sham/RDN	108 ± 13	100 ± 10	0.2222

Data are shown as mean ± SEM.

Supplemental Table 2. The primer sequences.

Gene	ID	Forward Primer	Reverse Primer
TNF-α	397086	AGCACTGAGAGCATGATCCGAGAC	TGCGACCAGGAGGAAGGAGAAGA
IL-1β	397122	GTGATGGCTAACTACGGTGACAACA	GCTTCTCCACTGCCACGATGAC
IL-6	399500	GCCTTCAGTCCAGTCGCCTTCT	TGGCATCACCTTTGGCATCTTCTTC
TGF-β	397078	GGAGCCTAGACACTCAGTACAGCAA	CAGGAACGCACGATCATGTTGGA
INF-γ	396991	TGGTAGCTCTGGGAAACTGAATGAC	TCTCTGGCCTTGGAACATAGTCTGA
IL-10	397106	GCAGCCAGCATTAAGTCTGAGAACA	GGTCAGCAACAAGTCGCCCATC
Col1A1	100738132	CTCAAGATGTGCCACTCCGACTG	GGTCTCGCCTGTCTCCATGTTG
Col3A1	100152001	CAGGAGGAGGAATCGGAGGCTATC	GGCACCAGGATGACCAGATACAC
GAPDH	396823	GCAAGTTCCACGGCACAGTCAAG	CTCGCTCCTGGAAGATGGTGATGG
