# Supplementary material for: Renal Denervation Attenuates Adverse Remodeling and Intramyocardial Inflammation in Acute Myocardial Infarction With Ischemia–Reperfusion Injury
Source: Front Cardiovasc Med. 2022 Apr 28;9:832014. doi: 10.3389/fcvm.2022.832014 (PMC9095912; doi:10.3389/fcvm.2022.832014)

Supplemental Materials

Supplemental Figure 1. Representative immunofluorescence staining of CD163 monocyte-macrophage in porcine heart IZ, BZ and RZ between two groups.

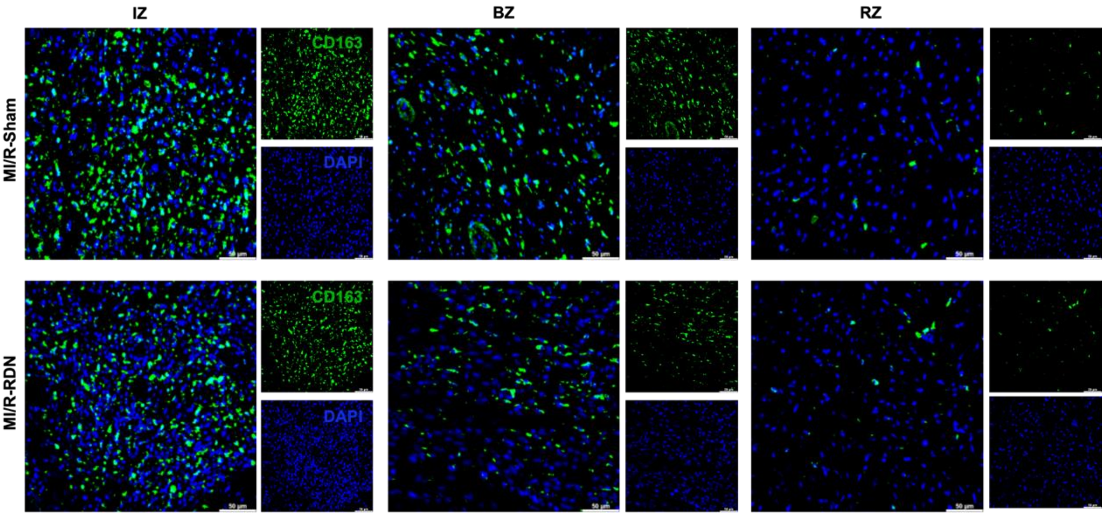

Supplement: Supplementary file 3 [file Image_1.PDF]
