# Supplementary figures and images for: Renal Denervation Attenuates Adverse Remodeling and Intramyocardial Inflammation in Acute Myocardial Infarction With Ischemia–Reperfusion Injury
Source: Front Cardiovasc Med. 2022 Apr 28;9:832014. doi: 10.3389/fcvm.2022.832014 (PMC9095912; doi:10.3389/fcvm.2022.832014)

## Supplemental Materials

### Uncropped gels

**Supplementary Figure 2.** Full unedited gel for Figure 6A.

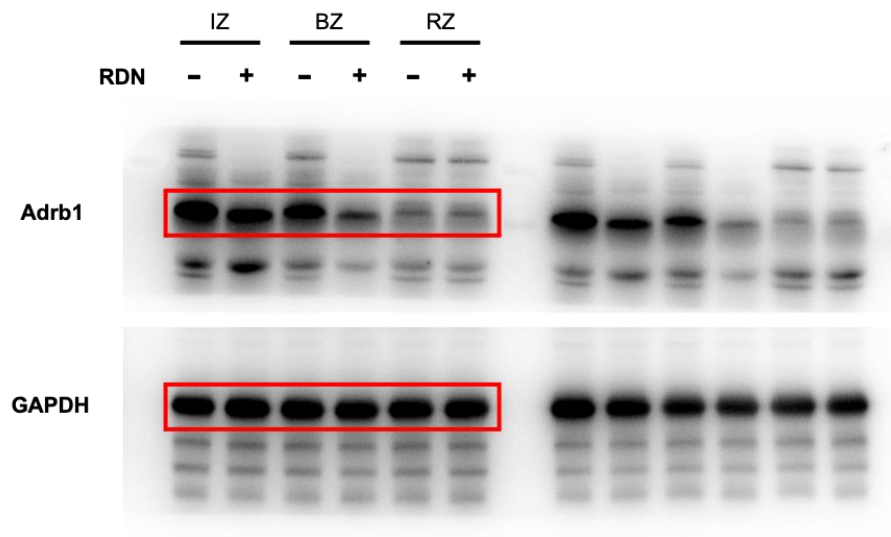

Supplement: Supplementary file 4 [file Image_2.pdf]
